# Real-time capture of reactive intermediates in an enzymatic reaction: insights into a P450-catalyzed oxidation†

**DOI:** 10.1039/d5sc02240a

**Published:** 2025-05-19

**Authors:** Pragya Pahchan, Abhijit Nandy, Eswarayya Ramireddy, Shibdas Banerjee

**Affiliations:** a Department of Biology, Indian Institute of Science Education and Research Tirupati Tirupati 517619 India eswar.ramireddy@iisertirupati.ac.in; b Department of Chemistry, Indian Institute of Science Education and Research Tirupati Tirupati 517619 India shibdas@iisertirupati.ac.in

## Abstract

Enzymes are indispensable for a myriad of biochemical reactions in nature. Although rapid spectroscopic techniques and *operando* methods allow for real-time assessment of enzyme kinetics, isolating and identifying the reactive intermediates in enzymatic reactions remains a significant challenge. These intermediates often possess fleeting lifespans, prohibiting their direct detection. This study harnessed online mass spectrometric techniques and microfluidic sampling methods to monitor the formation of reactive intermediates in real time during enzymatic catalysis *in vitro*. Using CYP175A1, a thermophilic P450 enzyme known for its dual-function capabilities in peroxygenase and peroxidase activities, we investigated the oxidative dimerization of 1-methoxynaphthalene. This biocatalysis involves multiple intermediates, including resonating radical forms. By transposing the enzyme reaction into a 500 mM ammonium acetate buffer and spraying it into a mass spectrometer, we could detect various transient intermediate species, suggesting their preservation in buffer droplets during their flight. The resonance-like radical intermediates were distinguished and temporally evaluated using a parallel reaction monitoring approach in tandem mass spectrometry after being trapped with TEMPO, a radical marker. This comprehensive analysis successfully elucidated the complete catalytic cycle of the enzyme, offering a robust method for gaining deeper insights into enzyme action by tracking multiple intermediates in real time.

## Abstract 2

A P450-catalyzed oxidation was studied using online mass spectrometry coupled with a custom-built microfluidic sampling setup, enabling real-time identification of multiple intermediates and providing valuable insights into the biocatalytic process.

## Graphical abstract

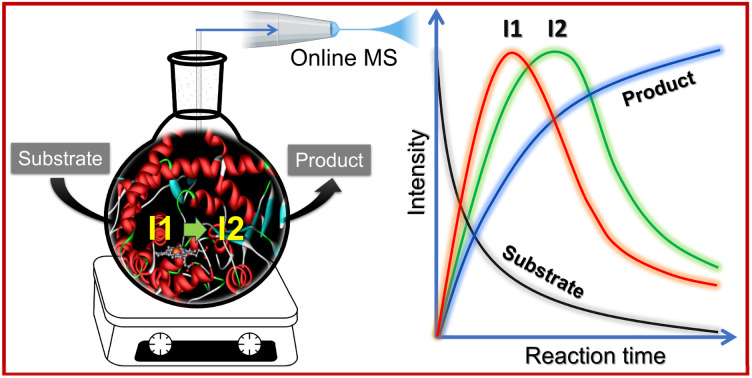

## Introduction

The *in situ* detection of reactive intermediates during enzymatic reactions is essential for understanding enzyme catalysis and its underlying mechanisms. Such reactive intermediates are usually short-lived and difficult to detect using conventional spectroscopic techniques, as their lifetimes often fall below the measurement timescale of most of the spectroscopic methods, or often those species are too low in concentration to be reliably detected.^1–4^ Despite these limitations, several enzymatic reactions have been explored through advancements in rapid spectroscopic techniques, aiming to capture the fleeting intermediate species. For example, Rittle and Green achieved the first direct spectroscopic characterization of compound **I** (highly reactive ferryl (Fe^IV^=O) porphyrin π-cation radical species) in cytochrome P450 using a combination of UV–vis, EPR, Mössbauer, and resonance Raman spectroscopy.^5^ Tosha *et al.* (2017) utilized time-resolved X-ray free-electron laser (XFEL) crystallography to capture an initial intermediate (nitric oxide-bound form of the enzyme) during the P450nor enzymatic reaction.^6^ A follow-up study by the same research group integrated flow-flash infrared spectroscopy with XFEL-based crystallography to capture the subsequent Fe^3+^–NHO˙^−^ radical intermediate, providing valuable insights into the radical–radical coupling mechanism underlying N–N bond formation during N_2_O generation in the P450nor reaction.^7^ Time-resolved nuclear magnetic resonance (NMR) spectroscopy has been employed to detect unstable, short-lived intermediates in enzymatic reactions. For instance, studies on acetyl-CoA synthetase have demonstrated the utility of NMR in monitoring transient species during catalysis.^8^ Several other investigators also employed custom-designed apparatus for effectively detecting and characterizing transient biocatalytic intermediates.^9–15^ While many of these studies have successfully detected individual or target-specific intermediates, simultaneous real-time observation of multiple intermediate species as they dynamically evolve within the enzymatic environment remains a largely unexplored frontier.^16–18^ Notably, intercepting multiple intermediates during enzymatic reactions presents a formidable challenge, particularly when the species are isomeric, resonance-like forms or lack distinct spectroscopic signatures. Indeed, while several rapid spectroscopic techniques can effectively monitor enzymatic reaction kinetics *in situ*,^18–29^ deconvoluting the resulting data to resolve individual intermediates remains challenging.

In this context, online (real-time) mass spectrometry (MS), although relatively underutilized in the study of enzyme catalysis,^30–32^ holds significant potential for probing complex, multistep enzymatic processes by pinpointing multiplex information on intermediate states. Unlike traditional endpoint analyses or discrete time-point measurements that provide only static snapshots of isolated reaction stages, real-time MS enables continuous, temporally resolved monitoring of chemical or biochemical transformations.^30–36^ This capability allows for the direct, high-resolution observation of the dynamic interconversion of multiple intermediate species, providing unique mechanistic insights into catalysis. It is particularly valuable for deciphering the temporal sequence of reaction events, offering a view of how multiple intermediates emerge, evolve, and progress toward product formation, which otherwise may remain hidden when discrete time-point measurements are considered.

Moreover, the inherent sensitivity of MS makes it exceptionally well-suited for systems involving intermediates at trace levels. By leveraging subtle variations in mass-to-charge (*m*/*z*) ratios and analyzing time-dependent shifts in ion abundances, real-time MS facilitates not only differentiation and relative quantification, but also structural elucidation *via* MS/MS of otherwise elusive species, providing an analytical advantage over conventional spectroscopic methods.

**Scheme 1:**
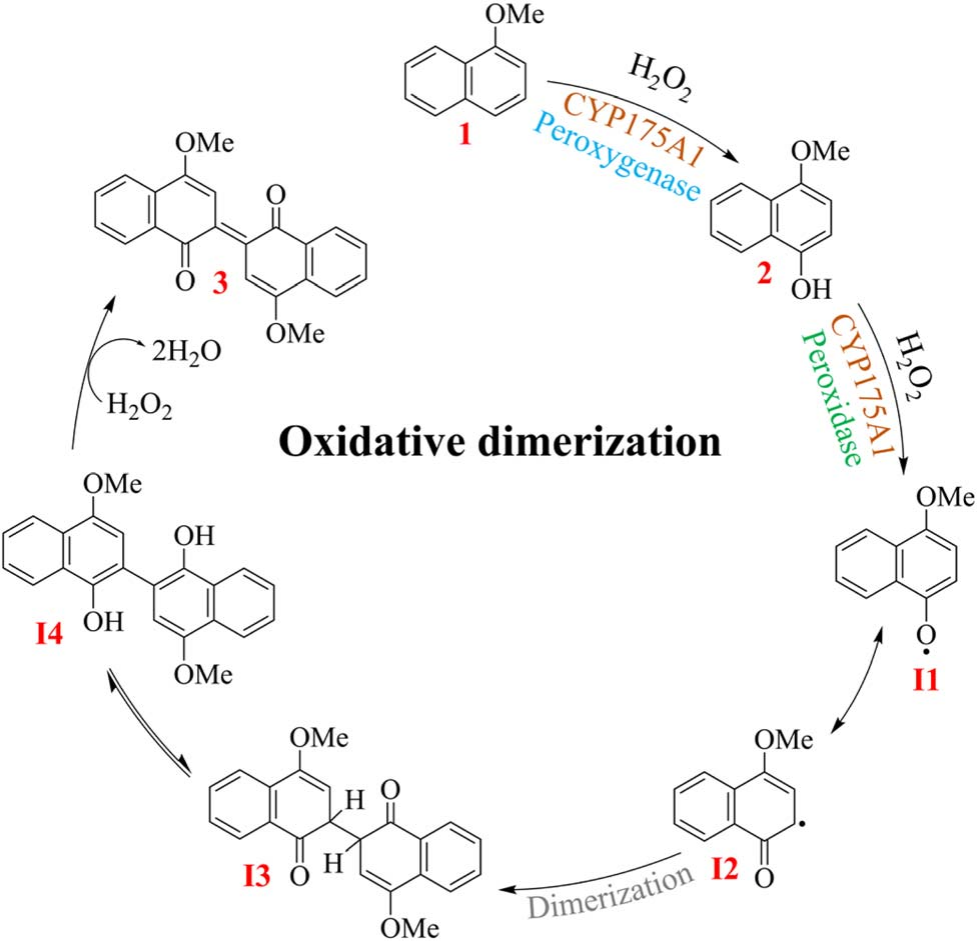
Proposed mechanism showing the dual activities (peroxygenase and peroxidase) of CYP175A1 in the oxidative dimerization of 1-methoxynaphthalene to the Russig's blue dye (3).

In view of this, we plan for the real-time monitoring of multiple intermediate species, including resonance-like forms, in typical dual-function enzyme catalysis, specifically focusing on the peroxygenase and peroxidase activities of CYP175A1.^37^ The thermostable nature of this bacterial (source: *Thermus thermophilus*) P450 enzyme,^38^ along with its broad substrate specificity,^39–45^ offers promising opportunities for its exploitation in the synthesis of value-added chemicals and pharmaceuticals under environmentally benign conditions.^46–48^ An earlier study revealed the biocatalytic activities of CYP175A1 on substituted naphthalenes in the oxidative pathway, demonstrating that the enzyme initially functions as a peroxygenase, converting these substituted naphthalenes into the corresponding naphthols.^37^ These naphthols then undergo *in situ* oxidative dimerization, possibly through the peroxidase-type activity of CYP175A1, forming dyes in various colors. The reaction is postulated to involve multiple intermediates that culminate in a dimeric product through oxidative aryl radical coupling, following a radical rearrangement reaction. For example, Scheme 1 presents the plausible mechanism of the CYP175A1-catalyzed oxidative dimerization of 1-methoxynaphthalene to 4,4′-dimethoxy-[2,2′]-bi-naphthalenylidine-1,1′-dione, commonly known as Russig's blue.^37,49^

This study has leveraged the capabilities of online mass spectrometry methods to detect and characterize five reactive intermediate species that sequentially emerge in the P450 reaction pathway during the synthesis of Russig's blue dye (Scheme 1). The intermediates were identified based on their distinct time-dependent abundance in high-resolution mass spectra, continuously recorded throughout the reaction, and further characterized using tandem mass spectrometry. Additionally, this study also introduces a parallel reaction monitoring approach using dual-channel infusion mass spectrometry to trap and distinguish between resonance-like radical intermediates, enabling a detailed elucidation of the reaction mechanism.

## Results and discussion

**Fig. 1:**
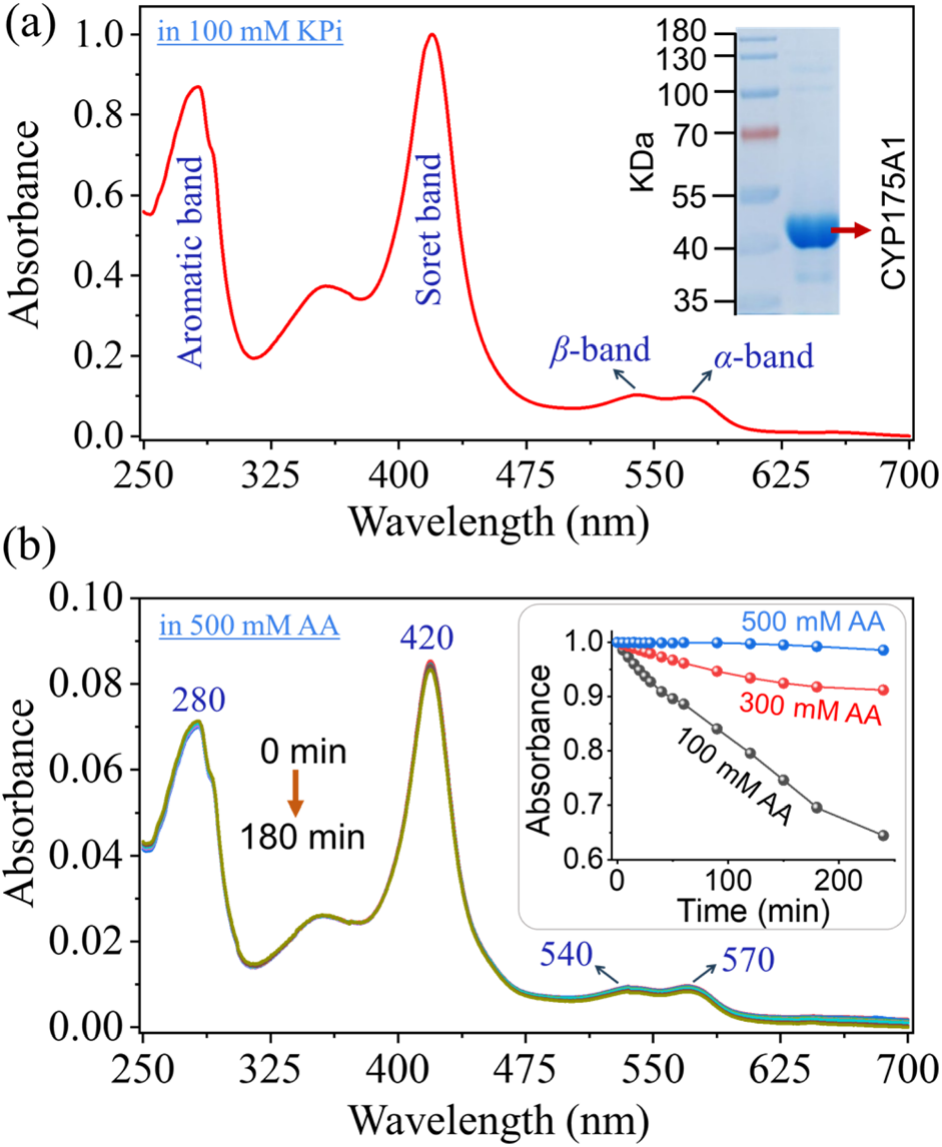
(a) Normalized UV-Vis absorption spectrum of the purified CYP175A1 fraction in 100 mM potassium phosphate buffer (pH 8). The inset displays an SDS-PAGE image indicating the enzyme purity. (b) Absorption measurements of CYP175A1 in 500 mM ammonium acetate (AA) buffer (pH 7.5) over a three-hour period as depicted by the different overlapped UV traces, indicating enzyme stability under these conditions. The inset shows the comparative stability of the enzyme in three different AA buffers, as indicated by changes in the Soret band.

**Fig. 2:**
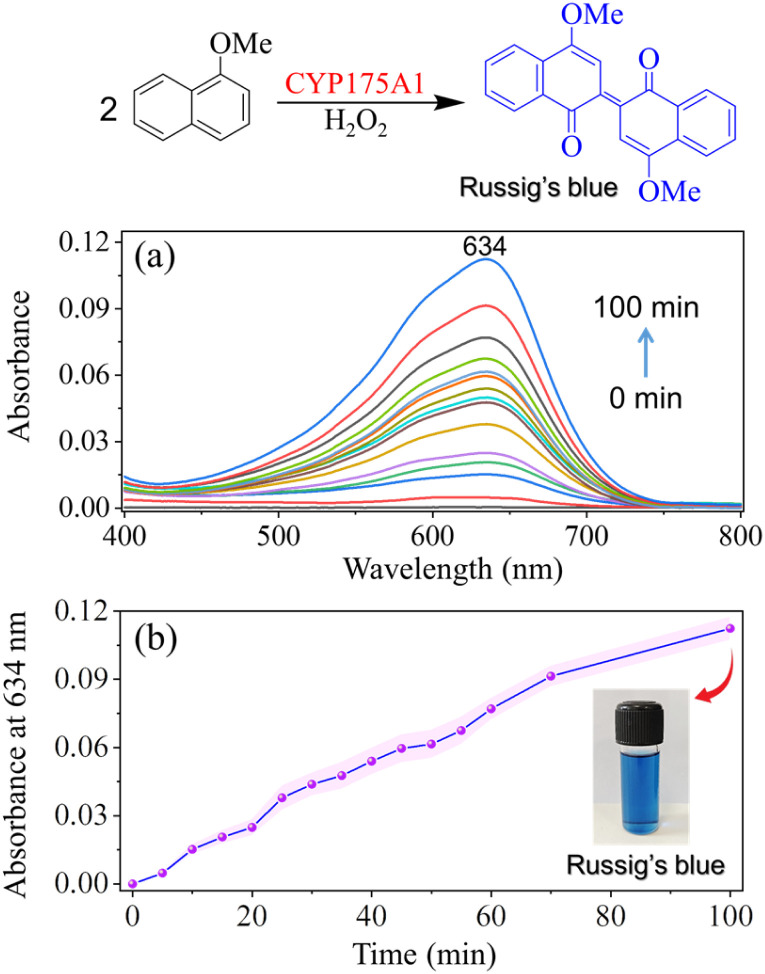
(a) UV-Vis monitoring of the CYP175A1-catalyzed formation of Russig's blue dye, indicating the increase in the absorption band of Russig's blue over the course of the reaction. UV-Vis spectra were obtained from the chloroform extract of the ongoing enzymatic reaction at various time intervals. (b) A plot showing the absorbance at 634 nm (derived from a) plotted against the reaction time. The inset displays a photograph of the blue dye extracted from the enzymatic reaction.

The thermostable P450 enzyme (N-terminal His-tagged CYP175A1) was expressed and purified according to a standardized protocol in our lab (see Materials and methods in the ESI†). The purity of the protein was assessed using UV-Vis absorption spectroscopy and SDS-PAGE (Fig. 1a). The UV-Vis absorption spectrum of the substrate-free P450 shows its characteristic Soret peak at 420 nm, with the β-band at ∼530 nm and the α-band at ∼570 nm, demonstrating the integrity of the protein and its association with the heme cofactor.^37,38,45^ The purified fraction of P450 was then subjected to a buffer exchange, replacing the potassium phosphate buffer (pH 8) with ammonium acetate buffer (pH 7.5), which is suitable for mass spectrometric analysis. We found that maintaining enzyme stability required a sufficiently high concentration of ammonium acetate (AA), such as 500 mM, as lower concentrations led to gradual enzyme degradation over time (Fig. 1b). The enzyme remained stable for an extended period, adequate for biocatalytic assays, in 500 mM AA buffer (pH 7.5) at room temperature, as determined by the UV-Vis study (Fig. 1b). The catalytic activity of CYP175A1 was then evaluated in the aforementioned AA buffer using 1-methoxynaphthalene as a representative substrate, which undergoes oxidative dimerization to produce Russig's blue dye (Scheme 1). The UV-Vis monitoring of blue dye formation, with an absorption maximum at 634 nm, confirmed that the reaction proceeds in the ammonium acetate (AA) buffer, albeit with slow kinetics (Fig. 2), consistent with the previously reported reaction in phosphate buffer (*k*_cat_ ∼ 9 min^−1^).^37^

**Fig. 3:**
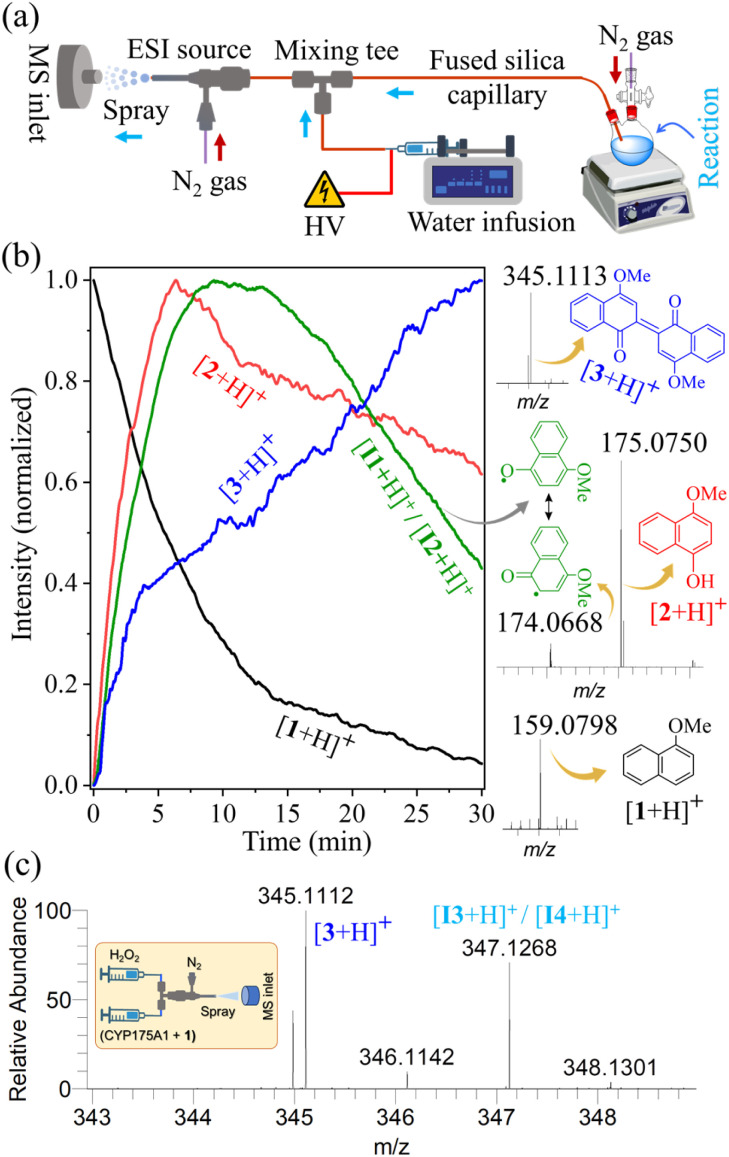
(a) Experimental setup with a custom-built pressurized sample infusion system for real-time mass spectrometric monitoring of the enzymatic reaction. (b) Extracted ion chronograms of species corresponding to the substrate (black curve), intermediates (red and green curves), and the product (blue curve), displaying their abundance (normalized to 1) over the reaction time. For clarity in presentation, only the selected ion signals of the species that form the basis of the reaction mechanism (Scheme 1), along with their corresponding structures, are displayed on the right. (c) The detection of intermediates I3/I4 was achieved by rapidly mixing the enzyme–substrate solution with H_2_O_2_ in a microfluidic tee-junction (see the inset), followed by the direct spraying of the reaction mixture into the mass spectrometer (Fig. S6†). All species were characterized by high mass accuracy (Table S1†) and MS/MS study (see the ESI†).

We used a custom-built pressurized sample infusion setup (Fig. 3a)^33–35^ in an online electrospray ionization mass spectrometry (ESI-MS) study to directly capture and detect the reactive intermediates and track their abundance in real time during enzyme catalysis. This experimental setup continuously delivered the reaction mixture, diluting it *via* a mixing tee, to a home-built electrospray source (Fig. S1†). The application of a high voltage (+5 kV) and nebulizing gas (110 psi back pressure) facilitated the electrospraying of the reaction mixture towards the inlet of a high-resolution mass spectrometer, enabling the detection of its chemical composition, including the substrate, intermediates, and final product (see Materials and methods for details). The reaction was initiated by injecting 40 μL of 250 mM H_2_O_2_ into a reaction vial containing 1 mM 1-methoxynaphthalene substrate and 5 μM CYP175A1 enzyme in 2 mL of 500 mM AA buffer, while the MS was continuously operated to detect the analytes right from the onset of the reaction. Our earlier studies indicated that reactive intermediates are stabilized at the air–water interface of microdroplets, particularly when the droplet surfaces are charged.^50–59^ Therefore, the enzymatic reaction aliquot confined within such electrospray generated microdroplets was expected to preserve some of the crucial intermediates, if not all, allowing for their reliable detection by high-resolution MS. Indeed, we identified the mass spectral signatures of all key intermediates, consistent with those postulated in the previously proposed mechanism (Scheme 1),^37^ along with the substrate and the dimeric product, as shown in Fig. 3b. This result confirms that CYP175A1 initially acts as a peroxygenase, converting 1-methoxynaphthalene (1) to 4-methoxy-1-naphthol (2), with the abundance of the latter reaching its peak in the reaction medium within 7 minutes of initiating the reaction. An MS/MS study unambiguously identified the species observed at *m*/*z* 175.0750 as protonated 4-methoxy-1-naphthol, the first intermediate of the reaction, which was successfully intercepted (Fig. S2†). Scheme 1 shows that 4-methoxy-1-naphthol could undergo oxidative dimerization through several intermediates (I1, I2, I3, and I4), mediated by the peroxidase-like activity of CYP175A1, to produce Russig's blue dye (3). This process was confirmed by separately using 4-methoxy-1-naphthol as the enzyme's substrate in a positive control study (Fig. S3†). The extracted ion chromatogram of a species detected at *m*/*z* 174.0668 was identified as the protonated forms of the resonance-like intermediates I1 and I2 (Fig. S4†). An ion signal at *m*/*z* 345.1113 was attributed to the formation of the dimeric product 3 (Russig's blue), which was further characterized by MS/MS (Fig. S5†). The extracted ion chronogram of all the aforementioned species obtained from the reaction medium during the reaction progression is presented in Fig. 3b, highlighting their time-dependent abundances. It is important to note that the reaction could be effectively monitored for up to 30 minutes, beyond which clogging of the MS inlet capillary interfered with data acquisition. Nevertheless, within this 30 minute window, more than 90% of the substrate was consumed, and the intermediates reached their maximum levels (Fig. 3b).

Although we were unable to detect the dimeric intermediates I3 and I4 (tautomers) using the pressurized sample infusion setup (Fig. 3a and b), likely due to their fast oxidation to product 3 in the presence of H_2_O_2_ in the reaction vial, we successfully identified these intermediates using a dual channel infusion setup (Fig. S6†).^56,58^ In this experiment, two solutions were independently infused at a flowrate of 2.5 μL min^−1^ into a mixing tee using two Hamilton syringes: one containing 5 μM CYP175A1 and 1 mM 1-methoxynaphthalene substrate in AA buffer, and the other containing 5 mM H_2_O_2_. Immediately after mixing, the reaction mixture was transferred through a short borosilicate capillary to the electrospray source, maintaining a distance of about 10 cm from the mixing tee to the MS inlet. This arrangement in the microfluidic reactor minimized the exposure time of intermediates I3 and I4 to H_2_O_2_, preventing their complete oxidation and enabling their rapid capture (Fig. 3c) and MS/MS characterization (Fig. S7†).

Given that aqueous microdroplets can promote or accelerate redox reactions,^57,60–64^ we conducted control experiments by electrospraying 1-methoxynaphthalene and 4-methoxy-1-naphthol in AA buffer containing H_2_O_2_ (Fig. S8†). However, none of these control studies resulted in the formation of Russig's blue dye in microdroplets. While we do not entirely rule out a partial contribution from microdroplet effects in the enzymatic reaction (Fig. 3c), the observed rise-and-fall pattern of the intermediate ion signal, along with the corresponding decrease in the substrate and increase in product species (Fig. 3b), closely mirrors the reaction profile associated with the bulk phase (reaction vial).

**Fig. 4:**
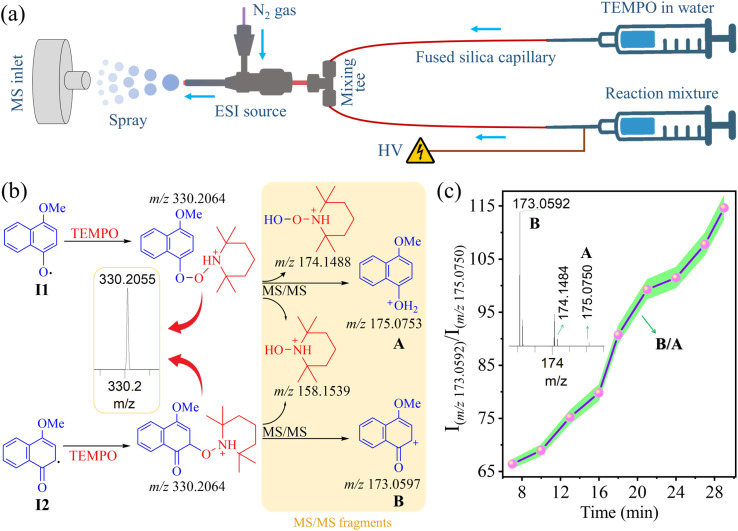
Detection of resonance-like radical intermediates. (a) Schematic of the experimental setup for mass spectrometric detection of TEMPO-trapped radical intermediates using a dual-channel infusion system. (b) Schematic presentation of MS detection for TEMPO-trapped oxyl radical (I1) and carbon radical (I2) intermediates, followed by their differentiation using tandem mass spectrometry. MS/MS fragments are highlighted in yellow with theoretical *m*/*z* values annotated beneath each fragment ion structure. (c) A plot illustrating parallel reaction monitoring of the transitions of two isomeric TEMPO-trapped intermediates (I1 and I2) into their distinct characteristic fragments (A and B), tracked through the B/A intensity ratio over the course of the enzymatic reaction. The green shaded regions represent experimental errors (standard deviation) from triplicate measurements. The inset shows ion signals of three typical fragments observed in the MS/MS data. All species were characterized by high mass accuracy (Table S2†).

As previously outlined (Scheme 1), the reaction is thought to occur *via* the intermediacy of the resonance-like I1 and I2 radicals, facilitated by the peroxidase-like activity of CYP175A1.^37^ Given that these protonated radical intermediates are indistinguishable due to their identical *m*/*z* values (Fig. 3b), we aimed to differentiate them by employing chemical trapping with TEMPO, followed by analyzing their MS/MS fragmentation over the course of the reaction. Fig. 4a illustrates a dual-channel infusion setup, analogous to Fig. S6,† for combining the reaction aliquot with TEMPO in the microfluidic system to trap the reactive radical intermediates for subsequent tandem mass spectrometric analysis. The peak at *m*/*z* 330.2055, shown in Fig. 4b, corresponds to the TEMPO adducts of the oxyl radical (I1) and carbon radical (I2) intermediates. These were isolated for the MS/MS study using high-energy collision dissociation (HCD). The analysis of characteristic fragments is shown in Fig. 4b. Employing a parallel reaction monitoring approach to track the transitions of the two isomeric TEMPO-trapped intermediates (I1 and I2) into their distinct characteristic fragments (A and B) demonstrated the gradual conversion of I1 to I2, as evidenced by the changing of the **B**/**A** intensity ratio during the enzymatic reaction (Fig. 4c). Two analogous experiments, one using 1-naphthylamine as a substrate (Fig. S9†) and the other using 1-naphthol (Fig. S10†), further validated this method for identifying distinct resonance-like radical intermediates generated during the biocatalysis of CYP175A1 on naphthalene derivatives. It is important to clarify that we refer to intermediates I1 and I2 as resonance-like species because they are real and distinguishable by mass spectrometry, in contrast to conventional canonical resonance structures, which are theoretical representations of instantaneous electron delocalization and cannot be individually observed. Nevertheless, this study successfully detected all reactive intermediates shown in Scheme 1, including the resonance-like forms of reactive radicals that underpin the reaction mechanism.

## Conclusions

While online mass spectrometry is frequently used for the real-time tracking of reactive intermediates in chemical reactions and for industrial/environmental analysis, it has rarely been applied to track intermediates in enzymatic reactions or biocatalysis. In this study, we harness the capabilities of online mass spectrometry to analyze an enzymatic (P450) reaction involving a range of reactive intermediates, some of which are particularly challenging to distinguish due to their resonance-like and tautomeric structures, while others are highly prone to oxidation prior to capture. By employing tailored, need-based sampling strategies, we successfully captured and characterized these intermediates, providing deeper insight into the enzyme's function *in vitro*. As P450 is a crucial enzyme class involved in drug and xenobiotic metabolism, bioremediation, and biocatalysis, understanding its biocatalytic mechanism is essential for elucidating how it converts its substrate into the final product. This *in vitro* study demonstrates promising insights in this regard. Although this study focused on a specific model enzyme, CYP175A1, the approach is not limited to this enzyme alone; it can be extended to investigate the catalytic steps of various other enzymes.

## Methods

### Materials

All necessary chemicals, solvents, and other materials were obtained from commercial sources. The CYP175A1 enzyme was expressed and purified in our lab, as detailed in the ESI.†

### CYP175A1 biocatalysis

The oxygenation/oxidation of substituted naphthalene by CYP175A1 was performed by incubating a 1 mL reaction mixture in a 4 mL reaction vial at 37 °C for 100 minutes, unless otherwise noted. The reaction mixture consisted of 5 μM CYP175A1, 1 mM α-substituted naphthalene (such as 1-methoxynaphthalene), and 5 mM H_2_O_2_ in the 500 mM ammonium acetate buffer (pH ∼ 7.5). Control reactions included all components of the assay mixture except for either CYP175A1 or H_2_O_2_. A kinetic study was conducted by analyzing the chloroform extract of the reaction at various time intervals (up to 100 minutes) using a UV-Vis spectrophotometer.

### Online ESI-MS study

A pressurized sample infusion setup (Fig. S1†), as originally described by McIndoe and coworkers,^33–35^ was connected to a custom-built electrospray ionization source for the continuous transfer of the enzymatic reaction mixture (during the progress of the reaction) to a high-resolution mass spectrometer (Orbitrap Elite Hybrid Ion Trap-Orbitrap mass spectrometer, Thermo Fisher Scientific, Newington, NH). The reaction was carried out in a 5 mL two-neck Schlenk flask, held at 37 °C, using a 2 mL reaction mixture composed of a 500 mM ammonium acetate buffer (pH ∼ 7.5) with 5 μM CYP175A1, 1 mM α-substituted naphthalene, and 5 mM H_2_O_2_. The reaction flask was pressurized with nitrogen gas at a backpressure of 4 psi to enable the reaction mixture to flow through a borosilicate capillary to a tee junction. At this point, the reaction mixture was combined with pure water (LC-MS grade) supplied at a rate of 15 μL min^−1^ with a Hamilton syringe. From the tee junction, the diluted reaction mixture was directed to a custom-built electrospray source through another borosilicate capillary for mass spectrometric analysis of the reactant, intermediates, and product. The spray source was constructed using a 1/16-inch stainless steel Swagelok tee that configured two coaxial capillaries (Fig. S1b†). The operation of the ESI source was enabled by the flow of the solution (from the mixing tee) through the inner fused silica capillary (100 μm i.d., 360 μm o.d.), while a coaxially aligned stainless-steel capillary (0.5 mm i.d., 1.6 mm o.d.) supplied nitrogen sheath gas at a backpressure of 110 psi. To optimize nebulization at the spray nozzle, the tip of the inner silica capillary extended 1 mm beyond the stainless-steel capillary's orifice. A high-voltage (+5 kV) DC potential was applied to the syringe needle to facilitate the electrospray process. The resulting stream of charged microdroplets from the nozzle was directed to the MS inlet capillary, maintained at 300 °C, ensuring efficient desolvation of the analyte ions for mass spectrometric detection. The maximum ion injection time for a single microscan was set to 500 ms, with the spray tip positioned 15 mm from the MS inlet. The mass resolution was set to 120 000, and ion optics parameters were fine-tuned to maximize the ion current. Data acquisition was performed using Thermo Fisher Scientific's Xcalibur software. The dead time, the period needed for the reaction mixture to travel from the reaction vial to the electrospray nozzle, was approximately 30 seconds and factored into the timing of the reaction onset. The high mass spectral accuracy provided confidence in the identification of reactants, intermediates, and products.

### MS/MS study

Tandem mass spectrometry (MS/MS) analyses were conducted on an Orbitrap Exploris 120 (Thermo Fisher Scientific) utilizing high-energy collisional dissociation (HCD). MS/MS spectra were obtained with an isolation width of 0.4 *m*/*z* for selecting the parent (target) species, followed by activation using normalized collision energy ranging from 30 to 70%. Nitrogen served as the collision gas. The mass resolution was maintained at 120 000, with an injection time of 500 ms, and each scan involved two microscans. To study the reactive intermediates, the enzymatic reaction was carried out in a 500 μL Hamilton syringe, with the reaction mixture being pumped at a flow rate of 5 μL min^−1^ directly to the custom-built electrospray source, positioned 15 mm from the MS inlet. Data acquisition for each analyte species was carried out for 1 minute using Xcalibur software (Thermo Fisher Scientific).

### Dual channel infusion for radical intermediate trapping

Two 500 μL Hamilton syringes, one filled with a 200 μM aqueous solution of 2,2,6,6-tetramethylpiperidine-1-oxyl free radical (TEMPO) and the other with the enzymatic reaction mixture as used in the online ESI-MS study, were pumped each at a flow rate of 5 μL min^−1^ through a narrow borosilicate capillary (100 μm i.d. and 360 μm o.d.) to a mixing tee. From there, the mixed solution was channeled through another borosilicate capillary (50 mm in length, 100 μm i.d. and 360 μm o.d.) to the custom-built electrospray source for mass spectrometric analysis of the TEMPO-trapped radical intermediates. The distance between the mixing tee to the MS-inlet orifice was maintained at 10 cm. All mass spectrometric experimental parameters remained the same as those used in the original online ESI-MS setup, unless specified otherwise.

## Data availability

The additional data supporting this article are included in the ESI.†

## Author contributions

SB designed the research; SB and ER supervised the research; PP and AN performed experiments and analyzed data; PP wrote the first draft of the paper and SB revised it.

## Conflicts of interest

There are no conflicts to declare.

## Supplementary Material

SC-OLF-D5SC02240A-s001
