# Supplementary material for: Real-time capture of reactive intermediates in an enzymatic reaction: insights into a P450-catalyzed oxidation
Source: Chem Sci. 2025 May 19;16(25):11322–30. doi: 10.1039/d5sc02240a (PMC12108963; doi:10.1039/d5sc02240a)
Supplement: SC-016-D5SC02240A-s001 [file SC-016-D5SC02240A-s001.pdf]

Supplementary Information (SI) for Chemical Science.
This journal is © The Royal Society of Chemistry 2025

Supplementary Information

Real-Time Capture of Reactive Intermediates in an Enzymatic Reaction: Insights into a P450-Catalyzed Oxidation

Pragya Pahchan,[a] Abhijit Nandy,[b] Eswarayya Ramireddy,[a,*] and Shibdas Banerjee[b,*]

[a]Department of Biology, Indian Institute of Science Education and Research Tirupati, Tirupati 517619, India

[b]Department of Chemistry, Indian Institute of Science Education and Research Tirupati, Tirupati 517619, India

*Corresponding authors:

Shibdas Banerjee, E-mail address: shibdas@iisertirupati.ac.in

Eswarayya Ramireddy, E-mail address: eswar.ramireddy@iisertirupati.ac.in

Sr. No.	Contents	Page Number
1	Supplementary Materials and Methods	S2 – S4
2	Table S1	S5
3	Table S2	S6
4	Fig. S1	S7
5	Fig. S2	S8
6	Fig. S3	S9
7	Fig. S4	S10
8	Fig. S5	S10
9	Fig. S6	S11
10	Fig. S7	S11
11	Fig. S8	S12
12	Fig. S9	S13
13	Fig. S10	S14

Supplementary Materials and Methods

Materials. The essential components for bacteriological culture, protein expression, and purification were sourced as follows: Luria-Bertani (LB) broth, LB agar, kanamycin sulfate, phenylmethylsulfonyl fluoride (PMSF), isopropyl *β*-D-1-thiogalactopyranoside (IPTG), imidazole, potassium chloride, lysozyme (from hen egg white), potassium phosphate dibasic anhydrous, glycerol, ammonium acetate, and sodium hydroxide were obtained from HiMedia, India. 5-Aminolevulinic acid hydrochloride, 1-methoxynaphthalene, 1-naphthylamine, 1-naphthol, and 2,2,6,6-tetramethylpiperidine-1-oxyl free radical (TEMPO), Coomassie brilliant blue R-250 were procured from TCI Chemicals, Japan. Iron (III) chloride anhydrous, potassium dihydrogen phosphate, and hydrogen peroxide (H_2O_2) were sourced from Merck. Triton™ X-100, Amicon ultra-15 centrifugal filter unit was purchased from Sigma-Aldrich. All LC-MS grade solvents for mass spectrometric analysis, chloroform, PageRuler™ prestained protein ladder (10 to 180 kDa), and HisPur™ Ni-NTA resin were procured from Thermo Fisher Scientific. PVDF membrane syringe filter (0.45 µm pore size, 25 mm diameter) and Hamilton syringes were procured from Chromatopak. Polyacrylamide gel electrophoresis (PAGE) setup and all essential chemicals for this was sourced from Bio-Rad unless mentioned otherwise. Millipore water was used for buffer preparation and other experimental needs as required

CYP175A1 Expression. The pRSFDuet-1 plasmid, encoding the gene for WT CYP175A1, was a gracious gift from Prof. S. Mazumdar (Tata Institute of Fundamental Research Mumbai). The above recombinant plasmid (containing the His-tagged CYP175A1 gene) was subjected to the transformation into *Escherichia coli* (*E. coli*, BL21-DE3 codon plus RP cells) following the heat-shock method. The transformed cell suspension was spread onto an LB agar plate containing kanamycin as the selection antibiotic and then incubated overnight at 37°C to develop the bacterial colony. To initiate primary cultures, several colonies were inoculated into 10 mL of Luria-Bertani (LB) broth, each containing 1 mM of kanamycin, and incubated overnight. These primary cultures were then used to inoculate 700 mL of LB broth in a 2L flask, creating the secondary culture. When the optical density (OD at 600 nm) of the secondary culture reached 0.6-0.8, protein expression was induced by adding 1 mM isopropyl-*β*-D-thiogalactopyranoside (IPTG), a synthetic allolactose analogue. Additionally, 0.5 mM of 5-aminolevulinic acid (δ-ALA) and 5 mg/mL of ferric chloride were added as precursors for the protein's heme prosthetic group.

The growth conditions for the cells included LB broth (composed of 10 g tryptone, 5 g yeast extract, and 10 g NaCl per liter), with an addition of 1mM Kanamycin. During the primary culture phase, the cultures were incubated at 37°C with agitation at 220 rpm, while in the secondary culture phase and post-induction, the media was stirred at a reduced speed of 150 rpm at 37°C unless otherwise stated. After a 12-hour induction period, the cells were harvested by centrifugation at 6000g for 15 minutes at 4°C, and the resulting cellular pellet was stored at -80°C for future use.

CYP175A1 Purification. The cell pellet was resuspended in lysis buffer (8 mL/g) consisting of 50 mM potassium phosphate (pH 8.0), 300 mM sodium chloride, 0.1 mM PMSF, 1 mg/mL lysozyme, 0.1% Triton X-100, 5% glycerol, and 20 mM imidazole. The cells were then disrupted by stirring at 4°C for 60 minutes, followed by ultrasonication at 0°C using a Vibra-Cell Ultrasonic Processor. The ultrasonication regimen included a sequence of 2-second pulses on and 3-second pulses off, repeated for two cycles of 3 minutes each at 40% amplitude. The supernatant resulting from centrifuging the sonicated suspension at 57,000g for 90 minutes at 4 °C was purified using immobilized metal affinity chromatography (IMAC) on an ÄKTA start protein purification system equipped with a 1 mL HisTrap high performance prepacked column. The column was initially equilibrated with 10 mL of wash buffer 1 (100 mM potassium phosphate, 300 mM NaCl, 20 mM imidazole, and 5% glycerol). The syringe-filtered lysate was then loaded onto the column, followed by a wash with 70 mL of wash buffer 2 (100 mM potassium phosphate, 300 mM NaCl, 50 mM imidazole, and 5% glycerol). Subsequent washing used 40 mL of wash buffer 3 (100 mM potassium phosphate, 300 mM NaCl, 80 mM imidazole, and 5% glycerol), leading to elution with three distinct elution buffers (EB1, EB2, EB3), each containing increased concentrations of imidazole (120 mM in EB1, 150 mM in EB2, and 300 mM in EB3) while maintaining 100 mM potassium phosphate, 300 mM NaCl, and 5% glycerol. Elution volumes were 25 mL for EB1, 30 mL for EB2, and 20 mL for EB3. Following protein elution, the column was extensively cleaned with a buffer containing 500 mM imidazole, 100 mM potassium phosphate, and 10% glycerol.

Preservation of CYP175A1. The 50% glycerol stock of the protein was prepared by concentrating the pure elution fractions using an Amicon Ultra-15 centrifugal filter unit. The concentration process involved several steps: initially, the filter was rinsed with 30 mL of MilliQ water (MQW) to clean it, followed by equilibration with 30 mL of buffer (pH ~8.0) containing 100 mM sodium phosphate and 300 mM NaCl. Subsequently, all pure fractions

were combined and concentrated to a final volume of 800 μL to 1 mL through centrifugation at 3800g at 4°C. The concentrated enzyme solution was then mixed with an equal volume of glycerol to produce the 50% glycerol stock. After concentration, the filter was washed with the aforementioned buffer to remove any residual proteins, followed by a rinse with 30 mL of MQW to eliminate salts. The filter was then stored at 4°C in MQW. For mass spectrometry analysis, the 50% glycerol stock of CYP175A1 underwent buffer exchange using a PD10 column with 500 mM ammonium acetate buffer (pH ~7.5), ensuring the enzyme's stability at room temperature for at least 3 hours.

SDS-PAGE. The purified protein was subjected to sodium dodecyl sulphate-polyacrylamide gel electrophoresis (SDS-PAGE) to access the purity profile and molecular weight of the protein. The polyacrylamide gel consisted of 10% resolving gel and a 5% stacking gel. Molecular weight calibration was achieved using PageRuler™ Prestained Protein Ladder (10 to 180 kDa). The Coomassie brilliant blue R-250 staining was applied for protein visualisation following electrophoresis. Gel images were captured using a BIO-RAD Gel Doc XR+ Imager. The molecular weight and the extent of purification of the CYP175A1 was determined based on the appearance of the 46 kDa band.

UV-Vis study. UV-Vis absorption analyses were conducted using a quartz cuvette with a 1 cm path length on a Shimadzu UV-1900i spectrophotometer, spanning a wavelength range of 250-700 nm with a resolution of 1 nm. The protein CYP175A1 was only subjected to analysis when its Reinheitszahl (R_z) value was ≥ 1.1 ($R_z = A_{420\ nm}/A_{280\ nm}$), indicating high purity. This level of purity for the His-tagged protein CYP175A1 was further confirmed by SDS-PAGE analysis, as noted above. The presence of characteristic Soret absorption at approximately 420 nm, along with β-band at about 530 nm and α-band at around 570 nm in the UV-Vis spectrum, also verified the quality of purification when R_z was ≥ 1.1. The concentration of CYP175A1 was determined spectrophotometrically based on the absorbance at 420 nm, using a molar extinction coefficient (ε_{420}) of 104 mM^{-1} cm^{-1}. Additionally, the concentration of H_2O_2 was assessed spectrophotometrically using its molar extinction coefficient of 39.4 M^{-1} cm^{-1} at 240 nm.

Table S1. Listing of mass (m/z) accuracies for species detected in the online pressurized sample infusion mass spectrometric study (Fig. 3).

Species	***Detected as***	***Theoretical m/z***	***Observed m/z***	***Mass Accuracy (ppm)***
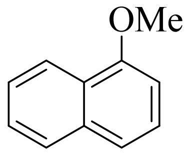	$[M+H]^+$	159.0804	159.0798	-3.77
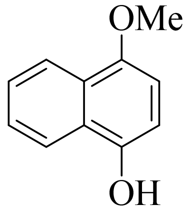	$[M+H]^+$	175.0753	175.0750	-1.71
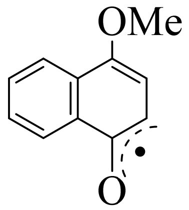	$[M+H]^+$	174.0676	174.0668	-4.5
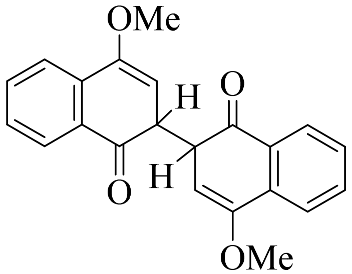	$[M+H]^+$	347.1278	347.1268	-2.88
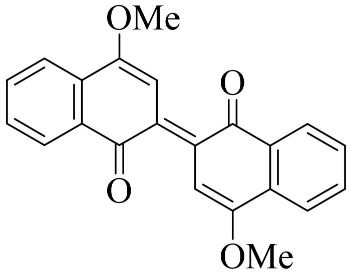	$[M+H]^+$	345.1121	345.1113	-2.32

Table S2. Listing of mass (*m/z*) accuracies for species detected in the radical trapping experiment (Fig. 4).

Species	*Detected as*	*Theoretical m/z*	*Observed m/z*	*Mass Accuracy (ppm)*
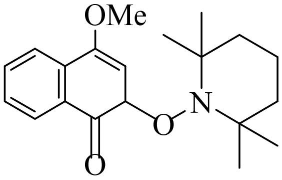	$[M+H]^+$	330.2064	330.2056	-2.42
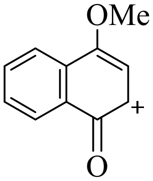	$[M+H]^+$	173.0597	173.0593	-2.31
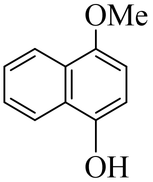	$[M+H]^+$	175.0753	175.0750	-1.71
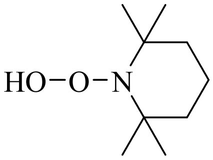	$[M+H]^+$	174.1488	174.1485	-1.72
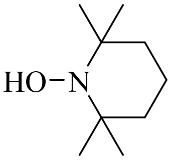	$[M+H]^+$	158.1539	158.1533	-3.79

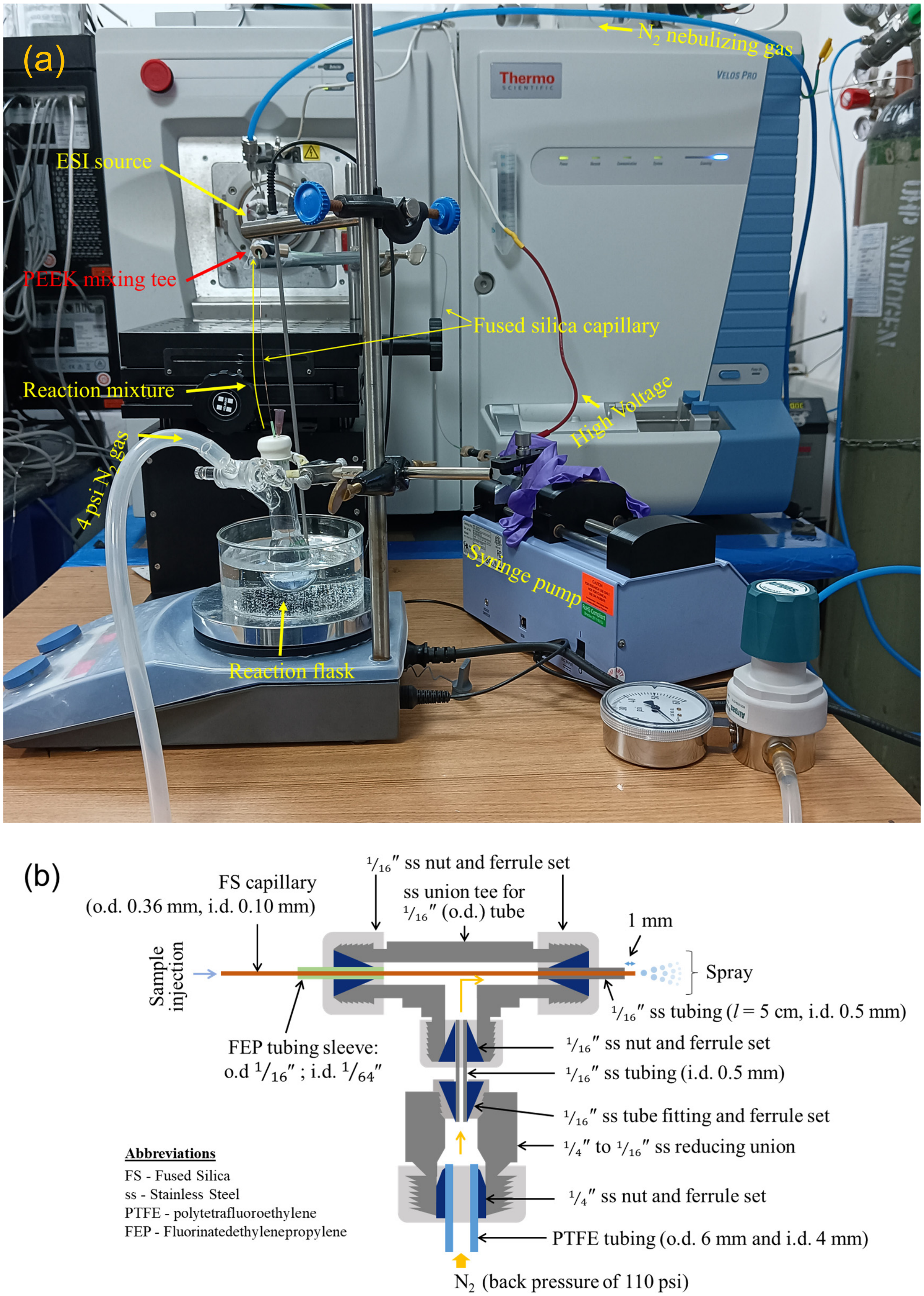

Fig. S1. (a) A photograph depicting the experimental setup, which includes the pressurized sample infusion system channelling the enzymatic reaction mixture (during the progress of the reaction) to the custom-built electrospray source, enabling simultaneous detection of the reactant (substrate), intermediates, and products. (b) A cross-section diagram depicting the construction of the electrospray source.

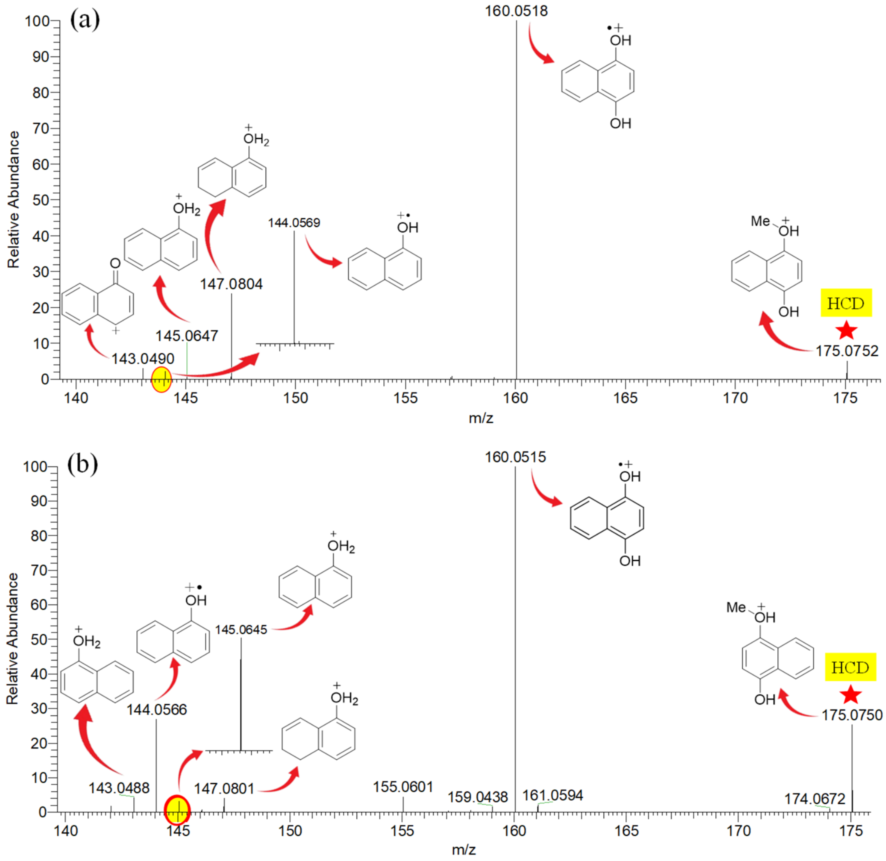

Fig. S2. HCD-MS/MS analysis was performed on the species with an *m/z* of 175.075 derived from (a) the reaction mixture and (b) a standard solution of 4-methoxy-1-naphthol in methanol. The fragment peaks are annotated with the likely structures, reflecting the structural integrity of the parent species.

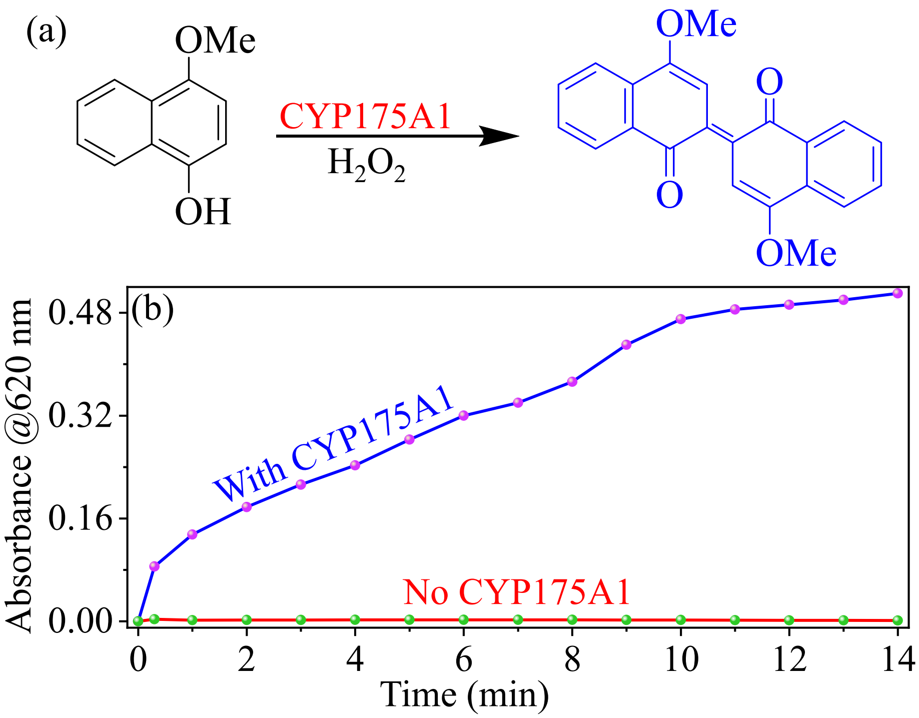

Fig. S3. Evaluating the peroxidase-like activity of CYP175A1 using UV-Vis spectroscopy. (a) A schematic depiction of the conversion of 4-methoxy-1-naphthol to Russig's blue (4,4′-dimethoxy-[2,2′]-bi-naphthalenylidine-1,1′-dione) catalyzed by CYP175A1 in the presence of H_2O_2. The reaction utilized 1 mM of the substrate, 5 µM of the enzyme, and 5 mM H_2O_2 in a 500 mM ammonium acetate buffer (pH ~7.5), conducted in a 1 mL glass vial at 37 °C. (b) A graph (blue line) illustrating the characteristic absorption at 620 nm of the product dye in the reaction mixture at different time points during the reaction's progression, with corresponding UV-Vis spectra recorded using a NanoDrop UV-Vis spectrophotometer (Thermo Fisher Scientific). Each spectrum was measured by aliquoting 2 µL of the reaction mixture for absorption analysis. No absorption at 620 nm was observed (indicated by the red line) in the reaction mixture containing the substrate and H_2O_2 but lacking the enzyme.

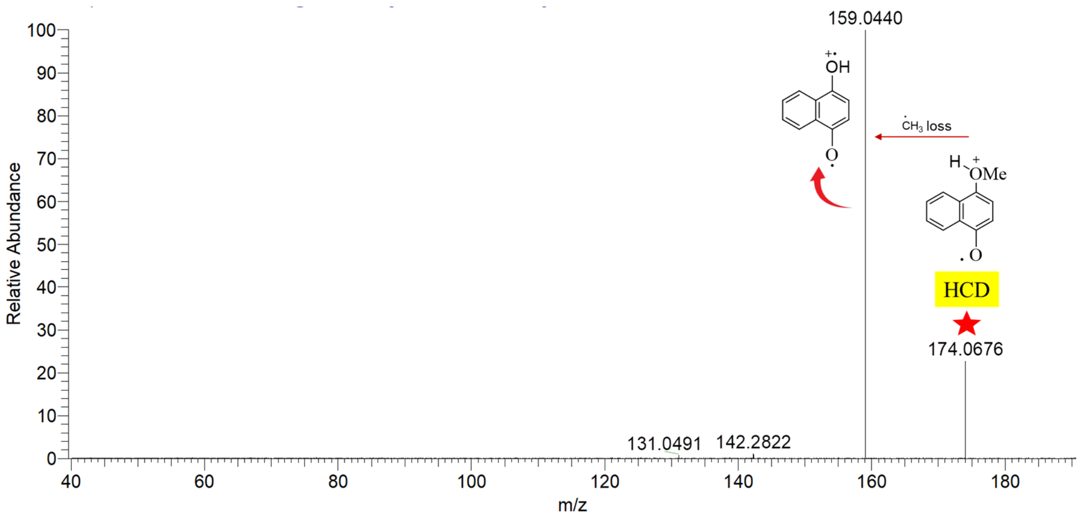

Fig. S4. HCD-MS/MS analysis of the species at *m/z* of 174.067 obtained from the reaction mixture. The characteristic fragments correspond to the [**I1/I2** + H^+] adduct. The fragment peak is annotated with probable structure, indicating the structural integrity of the parent species. The predominant fragmentation pattern, involving loss of a methyl radical, parallels that observed in the standard protonated 4-methoxy-1-naphthol (Fig. S2b). This similarity suggests that the species at *m/z* 174.067 includes a protonated methoxy group, and is likely associated with the reactive radical intermediates (**I1/I2**) as described in Scheme 1.

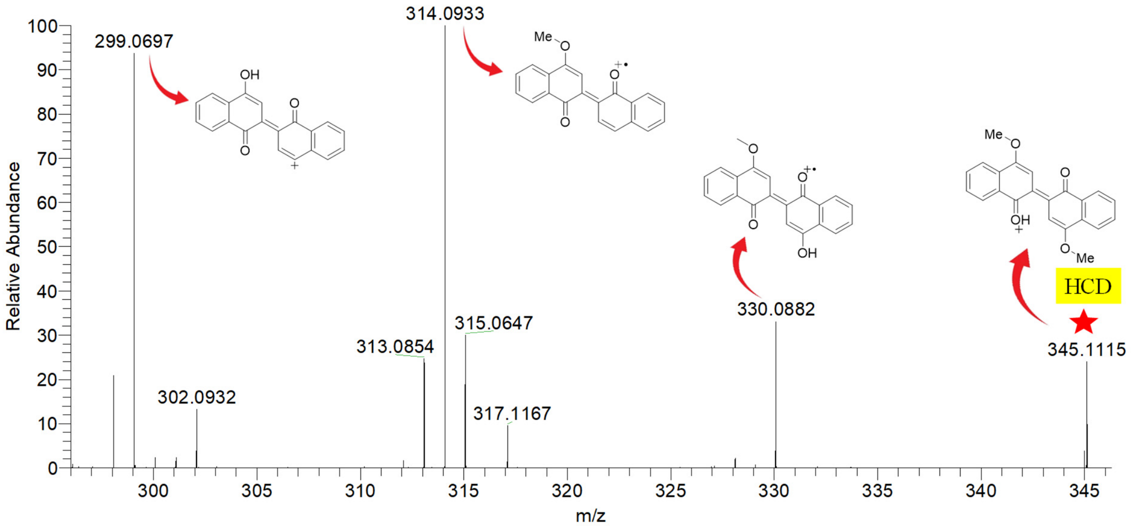

Fig. S5. HCD-MS/MS analysis of the species at *m/z* of 345.112 obtained from the reaction mixture. The characteristic fragments correspond to the protonated Russig's blue product [**3**+H^+]. The fragment peaks are annotated with probable structures, indicating the structural integrity of the parent species.

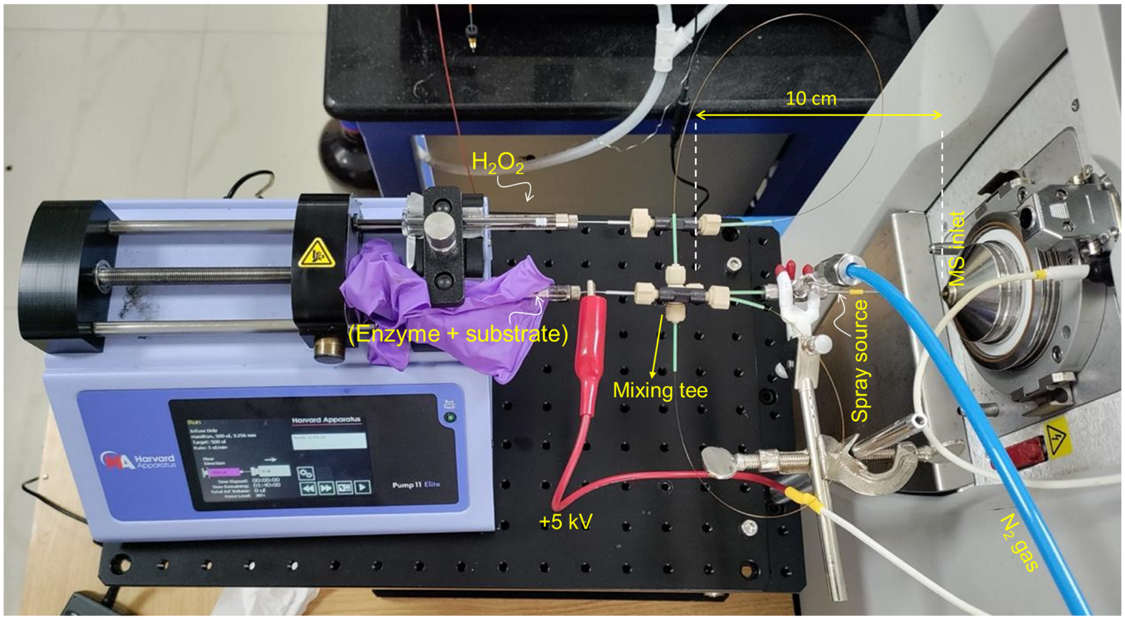

Fig. S6. Photograph of the experimental setup for the dual-channel infusion experiment. In this setup, a solution containing the enzyme and substrate was mixed with H_2O_2 at a mixing tee, followed by immediate transfer of the reaction mixture to the ESI spray source for detecting reactive intermediates. This method reduced the exposure time of the dimeric reactive intermediates (**I3** and **I4**) to the strong oxidant H_2O_2, allowing for their reliable detection (before they are fully oxidized to the product) through high-resolution mass spectrometry and MS/MS analysis.

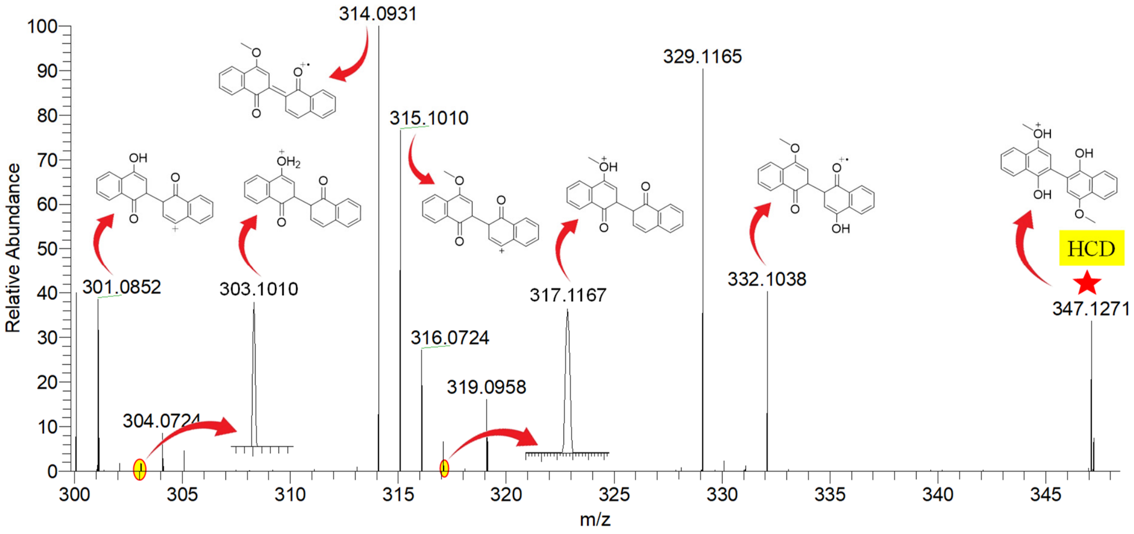

Fig. S7. HCD-MS/MS analysis of the species at *m/z* of 347.127 obtained from the reaction mixture. The characteristic fragments correspond to the [**I3**+H$^+$] or [**I4**+H$^+$] adduct. The fragment peaks are annotated with probable structures, indicating the structural integrity of the parent species.

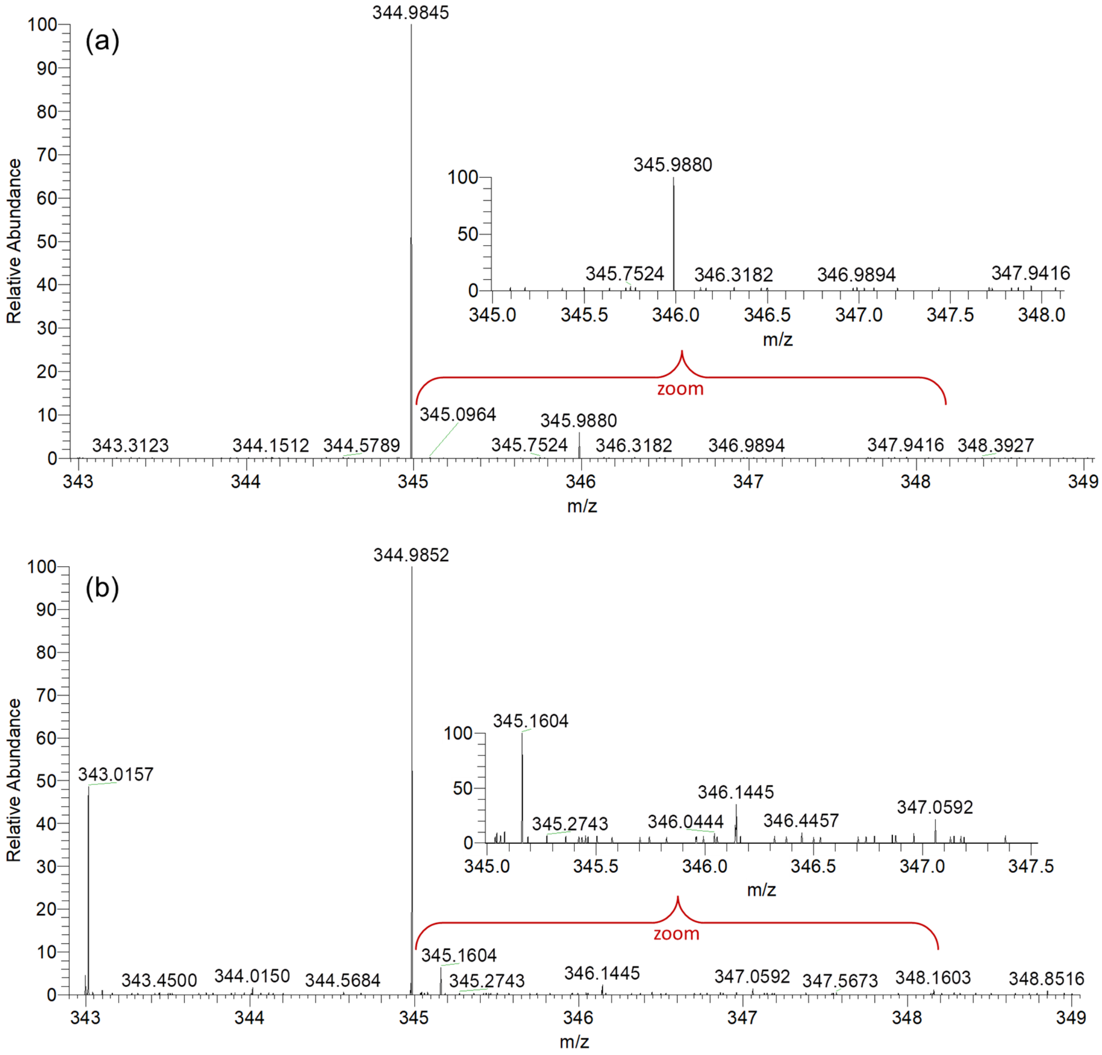

Fig. S8. Positive ion mode ESI mass spectra of (a) 1 mM 1-methoxynaphthalene in AA buffer containing 5 mM H_2O_2 and (b) 1 mM 4-methoxy-1-naphthol in AA buffer containing 5 mM H_2O_2. The spectral window shows no evidence of the Russig's blue product **3** or its precursor intermediates **I3** or **I4** (in contrast to Fig. 3), suggesting that aqueous microdroplets fail to promote the oxidative dimerization of either the starting substrate (**1**) or the hydroxylated intermediate (**2**).

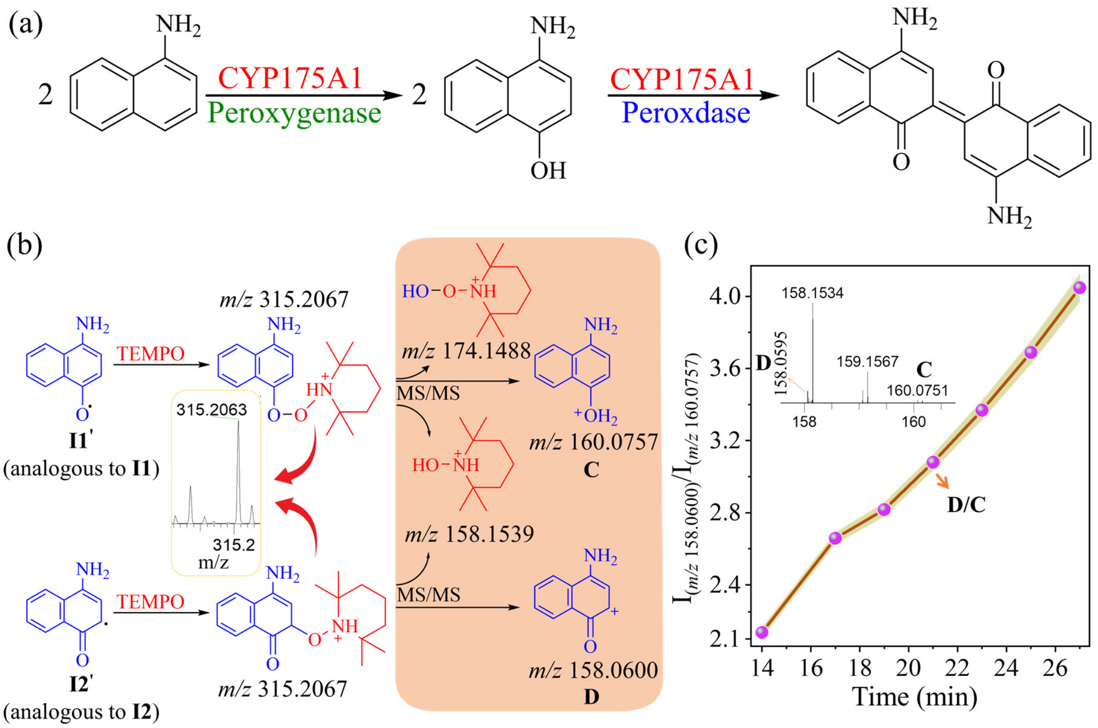

Fig. S9. Detection of resonance-like radical intermediates. (a) Schematic presentation of the CYP175A1-catalyzed oxidative dimerization of 1-naphthylamine, which is likely to follow the mechanism analogous to Scheme 1. (b) Schematic presentation of MS detection for TEMPO-trapped oxyl radical (**I1´**) and carbon radical (**I2´**) intermediates, followed by their differentiation using tandem mass spectrometry. MS/MS fragments are highlighted in yellow with theoretical *m/z* values annotated beneath each fragment ion structure. (c) A plot illustrating parallel reaction monitoring of the transitions of two isomeric TEMPO-trapped intermediates (**I1´** and **I2´**) into their distinct characteristic fragments (**C** and **D**), tracked through the **D/C** intensity ratio over the course of the enzymatic reaction. The green shaded regions represent experimental errors (standard deviation) from triplicate measurements. Inset shows ion signals of three typical fragments observed in the MS/MS data.

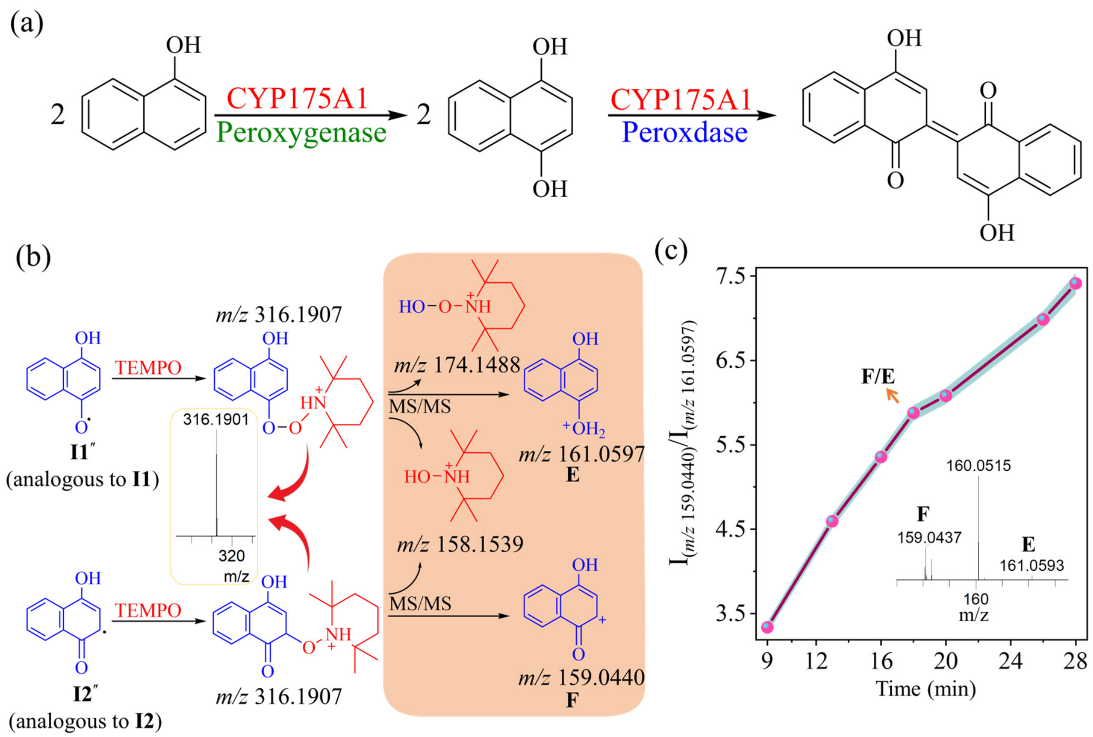

Fig. S10. Detection of resonance-like radical intermediates. (a) Schematic presentation of the CYP175A1-catalyzed oxidative dimerization of 1-naphthol, which is likely to follow the mechanism analogous to Scheme 1. (b) Schematic presentation of MS detection for TEMPO-trapped oxyl radical (**I1″**) and carbon radical (**I2″**) intermediates, followed by their differentiation using tandem mass spectrometry. MS/MS fragments are highlighted in yellow with theoretical *m/z* values annotated beneath each fragment ion structure. (c) A plot illustrating parallel reaction monitoring of the transitions of two isomeric TEMPO-trapped intermediates (**I1″** and **I2″**) into their distinct characteristic fragments (**E** and **F**), tracked through the **F/E** intensity ratio over the course of the enzymatic reaction. The green shaded regions represent experimental errors (standard deviation) from triplicate measurements. Inset shows ion signals of two typical fragments observed in the MS/MS data.
